# Mr. O'Neal's Case of Gout

**Published:** 1805-03-01

**Authors:** Hector O'Neal

**Affiliations:** Off Dungeness


*On receiving the following Case of Gout, treated on the cold Water Plan, we doubted whether the Author had correctly applied Dr. Kinglake's Mode of Treatment, and therefore a Copy was sent to Dr. K. for his Opinion, which is subjoined to the Case.*

---

### *To the Editors of the Medical and Physical Journal.*

Gentlemen,

Previously to entering upon the following Case, which I flatter myself you will think interesting, give me leave to premise, that I have always done my best endeavours to obviate the censure commonly thrown upon that class of the profession of which I have the honour to be a humble humble member, viz. that they are not acquainted with the modern discoveries in medicine, and the more recent modes of treating diseases. For my own part, I lay out all the money I can afford in the purchase of new publications; and I consider it my duty to try every improved mode of treatment that appears to me feasible, on the brave men entrusted to my care. On these principles I lately purchased the ingenious work of Dr. Kinglake; and being much pleased with the simplicity as well as the efficacy of his mode of curing the gout, that obstinate disease which can now no longer, with propriety, be styled the *opprobrium medicorum*, I resolved to put it in practice the first opportunity.

It was not long before an opportunity occurred of giving its efficacy a full and fair trial. The captain of our ship, a lieutenant in his Majesty's royal navy, about sixty years of age, who had been liable to attacks of the gout for these last ten years, after being exposed all night to a drizzling rain, in following the motions of some of the enemy's small craft along the French shore, complained in the morning of being sick at stomach. This he attempted to remove by some brandy and bitters, his usual remedy; but on this occasion it did not succeed. He had no appetite for dinner; and in the evening he called me into his cabin, and showed me his foot and ankle, which were considerably swelled and inflamed. I had no doubt of this being an attack of gout. Being determined to put the new practice to the test of experiment, I immediately immersed the foot and leg in a pail of fresh drawn sea water. (It may be necessary to inform the Reader, that the patient has only one leg, the other having been amputated just below the knee, on the glorious 1st of June). He at first objected to this plan, but soon found alleviation of the pain, and he agreed on going to his cot, to have the whole of the limb wrapped up in cloths wrung out of cold water. He slept well, but in the morning complained of a coldness in his stomach. On examining the leg, the redness and inflammation had quite disappeared. He wished for a glass of Madeira, or to have some brandy in his tea. I objected to both these requests, and being determined to give Dr. K's plan a fair trial, I had taken the precaution to have some water cooled by hanging it to the main stay in a bottle surrounded with wet cloths at an early hour in the morning, where it was exposed to the full effects of evaporation; of this I prevailed on him with some difficulty to drink a large glass. Conceiving that the the sense of coldness at his stomach, of which he still continued to complain, might be a gouty affection, I applied cloths wrung out of sea water to the patient's stomach, which were frequently renewed. During the day he complained of no pain, but he expressed no inclination for food. He frequently asked for some Madeira, or hot spirits and water; from these however, though with some difficulty, I prevailed on him to abstain. In the evening he seemed very heavy and much inclined to sleep. This I considered as a favourable symptom. About two o'clock, A. M. I was called out of my birth, and found my patient comatose, and affected with stertorous breathing. His face appearing very turgid and red, I took away, without loss of time, ten ounces of blood. But this not immediately rousing him, I applied cloths wrung out of salt water to the whole of his head, having previously taken off his night cap; and I desired my mate, who sat up with him, to keep them cool by pouring water from time to time on them from a can filled expressly for that purpose. He waked me about six, A. M. to say he was alarmed about the captain's situation. I found him insensible, with his eyes open, and breathing very hard. I had now no doubt of this being an apoplectic attack. I immediately opened the temporal artery, which however bled but little, and the patient expired about ten o'clock.

Now, Gentlemen, I would consider it as a favour, if you, or still more so, if Dr. Kinglake himself, would condescend to declare, for the satisfaction of some of our officers, who have been carping at this new mode of practice, and for the justification of my own conduct, whether any part of the plan of treatment I have now detailed, can be considered as having had any tendency to accelerate the death of the patient. For my own part, I have no doubt of the complaint having been apoplectic from the beginning, and that nothing could have prevented its fatal termination. I can hardly believe that wetting the cloths with salt water, instead of fresh, could have any material influence. On this point, also, I would humbly crave Dr. K.s kind opinion.

If you grant this insertion, you may depend on receiving an accurate account of my success in the next case of gout that falls under my care; being determined to follow up the same plan of treatment, till I see good reasons for laying it aside,

